# A Friendly Alias

**Published:** 1887-07-23

**Authors:** 


### A Friendly Alias.

By W. J. E.

(*Continued from p. 271, vol. ii.*)

Chapter III.—Altered Lives.

There were several smears, tear-marks, as Cyril conjectured, and in places the writing was faint and undecided. He was much touched by the perusal of it. After he had finished, he handed it to Heathcote in silence, and stared in the grate for some moments. Then he said:

"And that is Miss Seaton! A true Miranda, if ever was one. I think that if Cyril Clayton had met a girl like that, there would have been a Mrs. Clayton here to-night. Well! well!"—here he looked round the room—"I'm very comfortable." He sighed, and then added:

"And yet you wished to sever the connection!"

Heathcote answered him sadly and slowly: "Well! I'm glad I've made a clean breast of it to you. You can't tell what a girl is till the occasion tests her. I never knew Kirsty till to-day—I own it. May she, after all, get a better lover. But don't speak of it any more now; I feel it too deeply. Let us change the subject."

They smoked in silence some minutes. Then Clayton spoke:

"I've not been idle in your behalf, though I said nothing. Clayton, Clayton, and Jarrow have a little influence, you know, in the shipping line. Jarrow does most of the work, I admit. And I'm glad my father took him into partnership, as well as me, before he died, though I had a fair drilling in my trade. All the time you were at Oxford I was up and down the river from Elswick to Tynemouth, in office or abroad. But two heads are better than one, especially when one is suffering from a tendency to be lazy from being too well off. It wouldn't do for me to put you in our office here. Such a course would entail inconvenience to us and you. I want you to be useful to us, and learn the business quietly, as it were, at the same time. Now, we started an agency in Havre some time back. You can speak French very well, I think. What do you say to a twelvemonth there with our agent, getting versed in the ways of our firm? Jarvis is a capital fellow, without officiousness. The salary—"

Heathcote sprang to his feet and clasped Cyril's hand,—"As long as it's bread and cheese, I don't care. Cyril, you've been a good fellow to me! You know I'm not good at making speeches. I'm grateful to you."

"There, that's all right," said Clayton, filling his pipe once more. "I was thinking of Tennyson's 'Ulysses.' What a fine poem it is!"

"And the lines you're thinking of," said Heathcote,—"are?"

And Clayton repeated:

"Tho' much is taken, much abides, and tho'

We are not now that strength, which in old days

Moved earth and heaven; that which we are we are,

One equal temper of heroic hearts

Made weak by time and fate, but strong in will

To strive, to seek, to find, and not to yield."

And Heathcote's eyes glittered with a proud resolve to emulate the untiring ambition of the great poetic hero.

Langley was not long in making his few necessary preparations, and setting out for Havre. Proud and sometimes moody as he was, and up to this time ill-fitted for a commercial career, he on the other hand possessed an equipment of determination and natural intelligence much to be envied. His gentlemanly address and his fluency in the French tongue were two considerable advantages. It was not long before the agent of Clayton, Clayton, and Jarrow at Havre reported that a very useful lieutenant had been sent him.

Cyril Clayton watched his friend's progress from afar with great interest, and even ran over to Havre once or twice to see him. On each occasion Cyril, who was in reality, though he affected to call himself idle, a shrewd, pushing Tynesider, discovered that his friend was making very quick advances in commercial acquirements, and so far he was pleased. He also noticed, however, that a gloom had settled on his face which should not have been there, a gloom not to be accounted for by business anxieties, where everything was going well. When he made a casual inquiry as to how Miss Seaton was, the answer came in a tone of sadness, tinged with a carelessness hardly real:

"She writes sometimes. I write seldom. Her letters show the same amiable, good girl. I'm afraid mine are not as interesting as they should be. There is no hope of my reopening the courtship. I still hold that my course was right. I am working hard, and if a regret now and then crosses my mind, it does not sap my energies. I cannot go back now my ships are burned. Please don't worry about me, old fellow."

And so the conversation would end.

Now all this did not deceive Cyril Clayton. He was a man who habitually professed small sympathy with the vagaries and self-made troubles of lovers. "As for myself," he sometimes remarked, "I don't go in for it." It was very provoking. He was a fine, tall fellow, not unaccomplished, a keen business man, and well off. Most of the Newcastle beauties—and there are beauties at Newcastle—were very pleased at Christmas dances to secure Mr. Clayton as a partner, and to that end exercised all the legitimate arts of modest maidenly attraction that experience and their elder sisters had taught them. Yet season after season went by, and although mammas remarked, "It's time that young Clayton got married," "young Clayton" still obstinately remained a bachelor.

If he professed small sympathy with lovers in general, he was firmly convinced that his friend Heathcote must marry Miss Seaton, that they were both strongly desirous of it in their hearts, and that he could remove the difficulties that stood in the way. He knew he must do whatever was to be done by strategy, or he would offend his friend's temper by interference and possibly put the match outside the pale of possibility.

Cyril Clayton meditated. As a result of his meditations, he announced his intention to Mr. Jarrow of taking a month's or six weeks' holiday. He was going salmon fishing. Having given minute directions about his letters, he looked out waders, reel, rod, gaff, and basket, donned a plaid suit of remarkable thickness and roughness, and was seen by his partner comfortably seated in a "first-class smoking" of a train which was slowly dragging its length out of the central station of "canny" Newcastle.

To return to Christina Seaton. The first paroxysms now long past, and being in a calm frame of mind, she could read the entry in the diary without tears. She was sad, but her sadness was of a gentle type, which made her more thoughtful for and more amiable towards others. She even assumed towards her sister Elsie a sort of kind, old maid, elderly sister air, which, coming from a comely girl in the very springtide of womanhood, caused the somewhat (so Kirsty thought) irreverent Elsie to laugh, dance round her in a wild sort of way, and finish by an attempt to swing her round in a galop.

It was understood in the family that the engagement was at an end, though not even to Elsie had Kirsty explained under what circumstances. Her mother was rejoiced because of Kirsty's common-sense, and her own wisdom in not trying to force on a dissolution.

"Very sensible of her indeed," she told a friend, "to break off with young Heathcote. He was very nice, but really, when he lost his money it wouldn't have done at all. But I didn't urge her to it. It's no use forcing girls of Kirsty's age. If they make up their mind to marry a man, and there's any opposition, wild horses won't drag them away, common-sense or no. But let them alone, and if they've any sense, they'll come round of themselves. Another cup of tea, Mrs. MacPherson?"

One day Elsie came dancing into her sister's room and burst out:

"Kirsty! Such a nice fellow!"

"Really, Elsie! Fellow?" interposed Kirsty, reprovingly.

"Gentleman, then," said Elsie, with a mock curtsey. "Such a nice gentleman has taken the Craigdalloch fishings. I saw him this morning. I was about halfway down the brae getting some flowers, and I caught his eye from the other side. He's tall, and wears a splendid moustache."

"Elsie! how you talk!"

"Facts are chiels that wunna ding," retorted Elsie, mimicking the Doric, chiefly because she knew Kirsty considered it "provincial," and not to be used. "Nae mair wull moustaches—till they're shaved off," she added, by way of a complete shock.

"And his name's Lely," she went on; "and Mrs. MacPherson's giving a garden party to-morrow; and there's the invitation." Then she proceeded to waltz round the room with arms akimbo.

"I do wish, Elsie, you'd cultivate a little more repose. What has this Mr. Lely to do with the garden party?"

"Why! of course he'll be there! What a splendid chance for you, Kirsty!"

"You know, Elsie, I have done with all that," said Kirsty, decisively.

"Going in for cultivating pug-dogs and playing whist, I suppose?" rejoined Elsie.

"Please don't tease me, Elsie!" said Kirsty, as she went to the window and looked out, a tear gathering in her eye, which Elsie did not see, for she rattled on:

"And I can have the stranger all to myself. That's all very well; but 'seniores priores,' as Miss Stuttgart used to say. Besides, I'm only a bread-and-butter Miss, so Tom says, and you'll be looking lovely in a 'ravishing ethereal costume,' as the *Herald* reporter will put it, if he can peep through the hedge."

"If you will persist in talking such nonsense," said Kirsty, with a quiver in her voice, "I shall go down to mamma." Elsie kissed her sister in an impulsive way and withdrew.

As Elsie prophesied, Mr. Lely was at the garden party; and for some reason or other, all other eligibles had to pale their ineffectual fires. They included two young ministers of promise, a doctor, the son of a local banker, and a few of lesser note. He was attentive to the old ladies, "so amusing" to the young ones, and even Miss Christina Seaton, to whom he was introduced at an early juncture, was obliged to pronounce him "entertaining but not frivolous." Elsie, with the privileges of nineteen, was very open in her expressions of admiration. He was altogether an unqualified success.

Elsie's girlish remarks opened up a new vista of ideas in Mrs. Seaton's maternal brain. By a curious coincidence, Mr. Lely seemed to fall in with Mrs. Seaton's wishes; and in a very short time from his first arrival he was a welcome visitor at that lady's house, "Gordon Brae." What with his wading across the river sometimes of a morning—to get at the fish better, he said,—calling on Mrs. Seaton of an afternoon, looking in for a game of tennis, or sitting on the lawn with the girls, raising his fine baritone voice as the golden sunset faded into silver twilight, it was only natural that Strathhaven should begin to speak of a new match for Miss Seaton.

Mr. Lely smiled to himself as he noticed how innocently Mrs. Seaton was playing into his hands, all the time she fancied she was doing wonders with her cards. The truth was that Mr. Lely, though contriving to be much in Kirsty's company, was at present rather sounding her, rather weighing her character, than making any advances towards a more intimate footing. Could anyone have seen, behind that mask of good-humour and general *bonhomie*, his face, and read his brain, he would have discovered that the problem he was working out was—"Given a lady of such and such predilections, to find out whether I can propose to her without any risk of being accepted."

Having apparently arrived at satisfactory data, Mr. Lely proceeded a step further, and laid siege, as he judged it, in a more formal way. He made Kirsty various presents with such graceful tact, that they admitted of no refusal; paid her marked, but not too obtrusive attentions; was most assiduous in paying his court to Mrs. Seaton, who became more and more charmed with him, especially that he was every day approaching nearer to the most delicate topic of all. She became a self-appointed *aide* in his suit. Towards Elsie, before others, he adopted a sort of prospective brother-in-law attitude, and, when alone with her, a less restrained confidence, which perfectly charmed her, and caused her to urge Kirsty to take up a less impregnable position.

(*To be continued.*)

